# Genetic Analysis of the Functions and Interactions of Components of the LevQRST Signal Transduction Complex of *Streptococcus mutans*

**DOI:** 10.1371/journal.pone.0017335

**Published:** 2011-02-22

**Authors:** Lin Zeng, Satarupa Das, Robert A. Burne

**Affiliations:** Department of Oral Biology, College of Dentistry, University of Florida, Gainesville, Florida, United States of America; Tulane University, United States of America

## Abstract

Transcription of the genes for a fructan hydrolase (*fruA*) and a fructose/mannose sugar:phosphotransferase permease (*levDEFG*) in *Streptococcus mutans* is activated by a four-component regulatory system consisting of a histidine kinase (LevS), a response regulator (LevR) and two carbohydrate-binding proteins (LevQT). The expression of the *fruA* and *levD* operons was at baseline in a *levQ* mutant and substantially decreased in a *levT* null mutant, with lower expression with the cognate inducers fructose or mannose, but slightly higher expression in glucose or galactose. A strain expressing *levQ* with two point mutations (E170A/F292S) did not require inducers to activate gene expression and displayed altered *levD* expression when growing on various carbohydrates, including cellobiose. Linker-scanning (LS) mutagenesis was used to generate three libraries of mutants of *levQ, levS* and *levT* that displayed various levels of altered substrate specificity and of *fruA/levD* gene expression. The data support that LevQ and LevT are intimately involved in the sensing of carbohydrate signals, and that LevQ appears to be required for the integrity of the signal transduction complex, apparently by interacting with the sensor kinase LevS.

## Introduction

As the major etiological agent of human tooth decay, *Streptococcus mutans* is particularly well-adapted to growth in oral biofilms, where the intermittent nature of human feeding presents the organisms with a “feast or famine” existence [1]. *S. mutans* extracts energy from a spectrum of carbohydrates almost exclusively through glycolysis, releasing lactic and other organic acids that are responsible for demineralization of the tooth. The organism also secretes a fructosyltransferase (*ftf*) enzyme that converts sucrose into a fructose homopolymer (fructan) that accumulates rapidly in oral biofilms [2] and functions as an extracellular storage compound [3]. These fructans can be hydrolyzed into free fructose by the action of a secreted exo-β-D-fructosidase enzyme encoded by the *fruA* gene [4], [5], which is inducible and under the control of catabolite repression. The FruA enzyme contributes to the pathogenic potential of *S. mutans* by allowing the organism access to a greater amount of carbohydrate over an extended period of time [6]. A gene for a second predicted β-fructosidase enzyme (FruB) is co-transcribed with *fruA*, but the growth characteristics and fructosidase activity of a *fruA* mutant do not differ from those of a *fruAB* deletion mutant.

Two-component signal transduction (TCST) systems, typically composed of a sensor histidine kinase and a response regulator, play critical roles in physiologic homeostasis, environmental adaptations and pathogenic processes by altering gene expression in response to a wide variety of stimuli [7]. Interestingly, a small but increasing number of TCST systems have been found to be associated with auxiliary factors that influence signal transduction [8], the majority of which remain uncharacterized. Transcriptional regulation of *fruA* is under the control of an unusual four-component system that consists of the histidine kinase LevS, the response regulator LevR, and two putative extracellular sugar-binding proteins, LevQ and LevT [9], which are members of the substrate binding proteins of the ABC superfamily. All four components of the LevQRST system are required for efficient transcriptional activation of the *fruAB* operon, as well as another operon located immediately downstream of *levTSRQ* that encodes a fructose/mannose-specific sugar phosphotransferase system (PTS) Enzyme II complex (*levDEFG*) [9]. Both fructose and mannose [10] can serve as inducing signals for the LevQRST complex and a purified, histidine-tagged LevR protein was shown to bind to the promoter regions of *fruA* and *levD in vitro* [9]. Regulation of *fruA* is also sensitive to carbon catabolite repression (CCR) [11], [12]. Although binding of the catabolite control protein A (CcpA) homologue of *S. mutans* to catabolite response elements in the *fruA* promoter region occurs [13], CcpA plays a secondary role in CCR of *fruA*. Instead, CcpA-independent CCR exerts dominant control of transcription of *fruA* and *levDEFG* when various preferred carbohydrate sources are available. CcpA-independent CCR primarily involves interactions between the seryl-phosphorylated form of the phospho-carrier protein HPr, the response regulator LevR and the major glucose PTS permease ManL (EIIAB^Man^), but the FruI fructose PTS permease (EIIABC^Fru^) and the LevDEFG permeases can also impact CCR of *fruA* [14].

At the time of the discovery of the LevQRST four-component system, there were three similar complexes identifiable in the genomes of other bacteria [9]. That number has increased to at least 6 in the last 4 years with the availability of new genome sequences, with similar operons now identified in *Streptococcus gordonii, Streptococcus sanguinis, Lactobacillus johnsonii, Lactobacillus salivarius, Clostridium acetobutylicum* and *Dorea longicatena*. Both *S. sanguinis* and *S. gordonii* have a *fruA* homologue and results from our lab have proven that the LevQRST system in *S. gordonii* functions similarly to that in *S. mutans* (Tong, Zeng and Burne, in press). We report here a genetic analysis of structure:function relationships in the LevQRST pathway using various deletion, insertion and amino-acid-substitution mutants. The results begin to reveal the function of members of this pathway, including their possible roles in substrate binding, stimulus perception and signal transduction.

## Results

### Localization of LevQ and LevT

LevQ is predicted (http://www.oralgen.lanl.gov) to be an extracellularly-localized sugar-binding protein of the ABC superfamily anchored to the cell by a transmembrane domain. LevT is annotated as a membrane-associated ABC type sugar-binding protein, with the possibility of residing both within and outside of the cytoplasm. LevS is a sensor kinase predicted to contain up to 5 transmembrane domains. LevR, the cognate response regulator of the signaling complex [9], has no predicted signal peptide or transmembrane domains (see Figures S1, S2, S3 for results of computer modeling).

**Figure 1:**
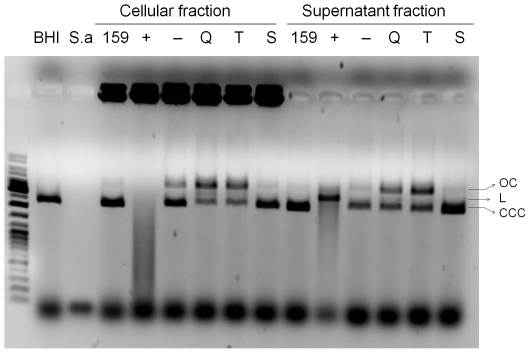
*In vitro* nuclease assays. Plasmid DNA (100 ng, pTZ18R) was incubated at 37°C for 1 h with the cellular or supernatant fractions of various *S. mutans* strains, followed by electrophoresis on an agarose gel. Positive controls: *S. aureus* (S.a.) and UA159/pVE8009 (+). Negative controls: fresh BHI medium (BHI), cultures from UA159 (159) and UA159/pVE8010 (−). Q, T, S: UA159 derivatives containing LevQ-Δ_SP_Nuc, LevT-Δ_SP_Nuc and LevS-Δ_SP_Nuc fusions, respectively. Open circular (OC), linear (L) and super-coiled (CCC) forms of the plasmid are labeled.

To test whether the sensor kinase and carbohydrate binding proteins could be exported from the cell, portions of the genes encoding the N-terminal segments of LevQ (up to Asp202), LevT (up to Asp72) or LevS (up to Ile253) were fused to the Δ_SP_Nuc sequence (Figure S4), which encodes a nuclease derived from *Staphylococcus aureus* that lacks its export signal [15]. Since the partial Nuc sequence has no signal peptide, its nuclease activity can only be detected outside of the host cells when it is fused to a polypeptide that is targeted for extracellular localization [15]. The fusion proteins were expressed from the cognate *lev* promoter on the chromosome and DNase activities from cell and supernatant fractions were tested in an *in vitro* assay using plasmid DNA as the substrate for the nuclease. In strains producing LevQ-Δ_SP_Nuc or LevT-Δ_SP_Nuc, plasmid-nicking or -cleavage activities were detected in both the supernatant fluid and the whole-cell fractions (Figure 1), suggesting that both LevQ and LevT are membrane-associated while their putative sugar-binding domains are targeted for the exterior of the cell. The reason for nuclease activity detected in the supernatant fluid of these samples was likely due to auto-cleavage of the fusion proteins caused by an internal peptide sequence of Nuc, which can result in release to the culture supernatant of mature NucA from cell surface [15]. Consistent with the notion that the histidine kinase domains generally function within the cytoplasm, the strain containing LevS-Δ_SP_Nuc fusion yielded no detectable nuclease activity in the cell-free extracts or in intact cells when assayed under the same conditions (Figure 1).

We have been able to generate a sufficiently high-titer rabbit antiserum against LevQ using a recombinant His-tagged LevQ protein fragment (excluding the first 39 amino acid residues) that was over-expressed in an *Escherichia coli* host. In contrast, when we used the same protocol to obtain an anti-LevS or anti-LevT antiserum, the reagents did not prove satisfactory for Western blot analysis. We believe this is partly due to the very low levels of expression of these proteins, coupled with the potential that they are comparatively unstable once the cell envelope has been disrupted. Still, using the anti-LevQ antiserum in an immuno-blotting assay, we detected strong signals of LevQ in samples homogenized in the presence of 5% sodium dodecyl sulfate (SDS) (data not shown).

**Figure 2:**
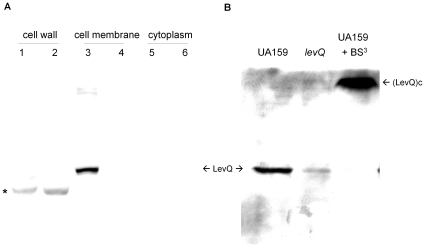
Western blots of LevQ protein generated using rabbit anti-LevQ antiserum. (A) Various fractions of T/ldh and Δ*levQ* culture were prepared from cells growing exponentially in BHI medium. 1, 3, 5: T/ldh; 2, 4, 6: Δ*levQ*. An asterisk indicates the non-LevQ immune-reactive band in cell wall preparations. (B) Whole-cell lysates were prepared by bead-beating with 5% SDS using cells of UA159, a Δ*levQ* mutant or UA159 treated with BS^3^. Both monomer and conjugates of LevQ (LevQc) are indicated by arrows.

To better determine the localization of LevQ, bacterial cell cultures were subjected to fractionation and samples derived from the cell wall, cell membrane and cytoplasmic fractions were collected and immune-blotted using anti-LevQ antiserum. Due to the low signal level of LevQ protein in the wild-type strain (Figure S5) and the presence of a non-LevQ cross-reactive species in the cell-wall-associated fractions, a *levQRST-*overexpressing strain T/ldh was constructed (see Materials and Methods). As shown in Figure 2A, the LevQ signal was only found in the cell membrane fraction and the cross-reactive protein in the cell wall fraction was not derived from LevQ (Figure 2A) or from the lysozyme/mutanolysin cocktail used to digest the cell walls (Figure S5). In addition, by applying a membrane-impermeable protein cross-linking reagent BS^3^ (bis[sulfosuccinimidyl] suberate) to *S. mutants* cells prior to homogenization using SDS buffer, we also successfully detected conjugates [(LevQ)c] containing the LevQ protein of higher molecular mass than that of monomeric LevQ (Figure 2). Interestingly, dimerization of LevQ could also be observed *in vitro* using a recombinant protein (Figure S6). Collectively, these results show that LevQ exists in a cell-associated form with its sugar-binding domain located outside of the cytoplasm.

### Impact of loss of LevQ or LevT

We previously reported that deletion of LevQ led to undetectable levels of expression from the *fruA* [9] or *levD* [10] promoters, as well as complete loss of growth when the β2,1-linked fructan polymer inulin, a substrate for FruA, was provided as the sole carbohydrate. In Table 1 we show that *levQ* mutant cells growing on all four of the tested sugars (glucose, fructose, mannose and galactose) produced little expression of the P*levD-cat* fusion [10]. Combined with our previous findings that deletion of the C-terminal sugar-binding domain of LevQ alone resulted in loss of function of this pathway [9], these results indicate that LevQ, and in particular its sugar-binding domain, play essential roles in the function of the LevQRST signal transduction complex.

**Table 1:** Expression of *levD* promoter:*cat* fusion as represented by the CAT specific activities in the wild-type strain UA159 and various *levQ* mutants.

Strain	CAT specific activity ± SD^a^ on various growth carbohydrates			
	Glucose	Fructose	Mannose	Galactose
***levQ^+^***	2.8±0.4	474.0±44.7	870.4±85.2	9.6±0.6
***levQ***	0.1±0.1	0.4±0.0	0.3±0.3	0.1±0.1
***levQ*** **con**	29.5±2.0	1.7±0.2	27.4±1.1	590.5±21.2
***levQ*** **con/** ***levR***	0.1±0.2	0.0±0.0	0.0±0.0	0.1±0.1
***levQ*** **con/** ***levT*** **ΔC**	26.0±1.8	1.2±0.4	8.0±2.4	547.8±25.4
***levQ*** **con/** ***levT*** **M1stop**	14.4±0.7	0.8±0.4	2.7±1.3	148.0±40.6
***levQ*** **E170A**	3.7±0.9	632.9±36.6	1,071.0±50.8	15.8±5.4
***levQ*** **F292S**	57.0±4.0	5.2±2.1	150.3±4.4	555.0±48.1
***levQ*** **LS35**	459.0±30.3	251.1±17.2	641.4±72.4	2,293.5±8.4
***levQ*** **LS46**	183.9±3.2	468.3±48.1	826.9±34.0	1,204.4±16.3

a The data are presented as the average results of three independent cultures. Cells were cultured in TV broth with 0.5% of the indicated carbohydrates and assays were performed as described in Materials and Methods. Activity is expressed as nmol of chloramphenicol acetylated (mg of protein^−1^×min^−1^).

To better understand the function of LevT, three mutants of the *levT* gene were engineered, including two mutants (*levT*ΔC) having an *em* or *sp* cassette inserted at the *Bam*HI site of the *levT* sequence and one point mutant (*levT*M1stop) engineered on the chromosome by substituting a translational stop codon (TAG) for the start codon (ATG) of the *levT* sequence (see Materials and Methods for detail). Based on the sequence of the insertion site and that of the antibiotic cassette, only the first 72 amino acid residues of LevT are expressed in these *levT*ΔC truncation mutants. The reason for using two different antibiotic cassettes was to ensure the transcription of downstream genes and to avoid marker conflicts in situations where we evaluated strains carrying multiple mutations. When the expression of the P*levD-cat* fusion was tested in these mutants growing on various sugars (Table 2), the two C-terminal truncation mutants behaved similarly, displaying poor *lev* gene expression on fructose or mannose. Also, twenty- to 60-fold higher levels of expression were observed in the mutants growing on galactose compared to that measured in the wild-type background, although expression levels differed by two- to three-fold between the two truncation mutants. The point mutant strain LevTM1stop, which should produce no LevT protein at all, had only 10 to 20% of the *levD* gene expression seen in the wild-type strain in the presence of the inducing sugars fructose or mannose, but displayed modestly increased (2.5-fold) expression when growing in galactose. Notably, the LevTM1stop strain behaved essentially the same as a strain of *S. mutans* in which the entire *levT* sequence was replaced by a *km* or *em* marker [9]. Furthermore, when growth on various carbohydrates was monitored, the LevTM1stop strain showed a small yet significant reduction in growth rate on fructose, but no change on glucose, and near complete loss of growth on inulin (Figure S7). Therefore, not only did complete loss of LevT alter the signal output from the two-component system, but changes in the response of the complex to cognate and non-cognate substrates was modified. In particular, the introduction of a truncated N-terminal version of this protein resulted in baseline expression of the LevQRST targets in the presence of inducing substrates, but higher levels of expression of these genes in the presence of carbohydrates that do not normally induce expression.

**Table 2:** Expression of *levD* promoter:*cat* fusion as represented by the CAT specific activities in the wild-type strain UA159 and various *levT* mutants.

Strain	Avg CAT specific activity ± SD^a^ on various growth carbohydrates			
	Glucose	Fructose	Mannose	Galactose
***levT^+^***	2.8±0.4	474.0±44.7	870.4±85.2	9.6±0.6
***levT*** **M1stop**	3.9±0.8	91.3±10.6	83.6±7.6	24.8±2.1
***levT*** **ΔC ** ***(sp)***	3.1±0.0	0.1±0.0	1.2±0.6	208.2±13.2
***levT*** **ΔC ** ***(em)***	6.4±0.9	0.7±0.4	0.6±0.1	592.3±36.8
***levT*** **ΔC ** ***(em)/levQ***	0.0±0.0	0.0±0.0	0.6±0.2	0.5±0.3

a The data are presented as the average results of at least three independent cultures. Cells were cultured in TV broth with 0.5% of the indicated carbohydrates and assays were performed as described in Materials and Methods. Activity is expressed as nmol of chloramphenicol acetylated (mg of protein^−1^×min^−1^).

### Point mutations in LevQ and LevT alter LevSR control of gene expression

A strain, designated LevQcon, carrying a mutated *levQ* gene was created using error-prone-PCR mutagenesis and was selected for further characterization based on a screen for isolates that showed increased expression of the P*levD-cat* fusion when growing on the normally non-inducing sugar galactose. Sequence analysis of the *levQ* gene in LevQcon identified 2 point mutations that resulted in replacement of a glutamic acid residue (Glu170) by alanine and a phenylalanine (Phe292) by serine. Expression of the P*levD-cat* fusion (Table 1) in the LevQcon background was markedly higher when cells were growing in non-inducing conditions, with 10-fold higher activity in TV-glucose and 60-fold higher activity in TV-galactose, compared with levels in the wild-type strain growing under identical conditions. Interestingly, much lower CAT activities were seen in the LevQcon background under inducing conditions, with cells growing on fructose showing 270-fold lower expression and those growing on mannose 30-fold lower levels than those expressed in the wild-type background. Therefore, the substrate specificity of the LevQRST signaling pathway could be altered by simple amino acid substitutions in one of the putative sugar-binding proteins. To help exclude the possibility that mutations extragenic to *levQ* were responsible for the observed phenotypes, the entire coding sequence of *levQ* in strain LevQcon was replaced by an erythromycin marker (*em*). The resultant strain behaved like *levQ* deletion mutants that were constructed independently (data not shown). To provide further proof that the behavior of the LevQcon strain was attributable to the identified changes in the LevQ protein, two separate mutants expressing LevQ with single amino acid substitutions that were present in the LevQcon strain, *levQ*F292S and *levQ*E170A (Table 1), were constructed as detailed in the methods section. The strains carrying the *levQ*F292S mutation closely resembled strain LevQcon, whereas *levQ*E170A differed only slightly from the wild-type strain in terms of expression patterns of the LevQRST-regulated genes. Thus, it appears that the *levQ*F292S mutation in LevQcon was responsible for the majority of the effects on gene expression. Additionally, growth tests showed that the LevQcon strain had a significantly reduced growth rate on fructose compared with the wild-type strain and loss of growth on inulin (Figure S7).

Interestingly, altered expression of the *levD* promoter in strain LevQcon was noted in nearly all carbohydrates tested (Table 3), including sucrose, sorbitol, melibiose, cellobiose, lactose and raffinose, compared to the wild-type background grown under identical conditions. Growth on melibiose resulted in higher levels (∼8 fold) of *levD* promoter activity than in the wild-type background, whereas the opposite effect (∼14-fold decrease) was seen during growth on lactose (Table 3). Both melibiose and lactose are disaccharides composed of galactose and glucose moieties, and both are utilized only after internalization through their respective transporters; lactose via a lactose-specific PTS and melibiose through the Msm ABC transporter [16], [17]. Notably, growth in cellobiose (glucose-β1,4-glucose), which is rapidly metabolized only after internalization by the PTS of *S. mutans* [18], elicited ∼45-fold higher *levD* expression than in the wild-type background.

**Table 3:** CAT specific activities representing the expression of P*levD:cat* fusion in the backgrounds of wild-type strain UA159, mutants *levQ*con and *levQ*con/*celB*.

Strain	Avg CAT sp act ± SD^a^ on various growth carbohydrates					
	Cellobiose	Lactose	Melibiose	Raffinose	Sorbitol	Sucrose
***levQ*** **^+^**	10.9±1.1	3.2±0.7	38.4±9.6	631.0±43.1	22.0±7.0	135.0±21.9
***levQ*** **con**	467.1±33.0	0.2±0.2	300.1±27.8	154.5±34.6	218.8±9.3	0.3±0.3
***levT*** **M1stop/TNP**	6.1±0.2	0.3±0.2	34.7±1.6	NT	12.1±0.6	NT
***levT*** **M1stop/** ***levT*** **LS13**	68.4±1.7	1.4±0.3	327.8±10.5	NT	708.7±8.5	NT
	Lactose	Lactose +5 mM Cellobiose		Lactose +20 mM Cellobiose		
***levQ*** **^+^**	3.2±0.7	0.6±0.1		0.7±0.2		
***levQ*** **con**	0.2±0.2	1.2±0.5		10.9±2.1		
***levQ*** **con/** ***celB***	0.6±0.5	0.5±0.4		7.1±1.6		

*^a^*The data are presented as the average results of at least three independent cultures. Cells were cultured in TV broth with 0.5% of the indicated carbohydrates and assays were performed as described in Materials and Methods. Activity is expressed as nmol of chloramphenicol acetylated (mg of protein^−1^×min^−1^).

NT, not tested.

The different expression levels from the *levD* promoter in LevQcon cells growing on the tested sugars could have arisen from differences in the affinity of the wild-type LevQRST and mutant LevQconRST signaling complex for the carbohydrates. Alternatively, differences in the rates of catabolism, or route of transport, of cellobiose, lactose and melibiose could influence *levD* promoter activity, since the *levD* operon is regulated by the PTS in response to energy levels in the cell [14]. To explore the possibility that the LevQconRST complex could perceive cellobiose as a signal substrate, the LevQcon strain was cultured in lactose to early exponential phase (OD_600_ = 0.1∼0.2), then different concentrations of cellobiose were added to the culture, cells were incubated for 3 h, and CAT assays were performed. Pulsing with 20 mM cellobiose clearly led to activation of gene expression through the mutant LevQconRST pathway (Table 3). In contrast, no induction of the *levD* promoter was detected when the experiment was performed in the wild-type (LevQRST) genetic background. It was also observed that a strain carrying the LevQcon and *celB* mutations, where CelB is the IIB component of the EII^Cel^ permease and *celB* mutants cannot internalize or metabolize cellobiose, displayed elevated expression of P*levD-cat* after pulsing with 20 mM cellobiose. Thus, it appears that extracellular cellobiose may trigger activation of the LevQRST complex in the LevQcon strain. In contrast to cellobiose, induction of *levD* expression by melibiose was not detected in strain LevQcon using the same type of test (data not shown). Therefore, it seems the effects of these non-cognate sugars on the LevQconRST complex can be attributed both to signaling through the complex and to effects on catabolite modification of *fruA/levD* expression by these growth substrates.

The epistatic relationship among the members of the LevQRST pathway was explored by introducing mutations in the *levR* or *levT* genes into the LevQcon strain. Replacement of *levR* with a spectinomycin (*sp*)-resistance marker in the LevQcon background led to baseline levels of expression of the P*levD-cat* fusion under all conditions tested (Table 1). Two forms of a mutated *levT* were introduced into the LevQcon strain separately; the *levT*M1stop mutation (*levQ*con/*levT*M1stop) and the *levT*ΔC C-terminal deletion with an *em* marker (*levQ*con/*levT*ΔC). Only minor differences were noted in the expression of P*levD-cat* in the *levQ*con and *levQ*con/*levT*ΔC backgrounds (Table 1). However, in strain *levQ*con/*levT*M1stop, lower CAT activities were expressed by cells growing on all sugars tested, relative to *levQ*con/*levT*ΔC or LevQcon. Although the molecular basis for these differences will require additional investigation, our results indicate that both LevT and LevQ are required for signal transduction by the complex, and that LevQ in particular has a profound effect on the substrate specificity of the system.

We also investigated the role of LevQ in the altered expression of the *levD* operon caused by the C-terminal truncation of LevT by mobilizing a *levQ* (*sp*) deletion into a *levT*ΔC (*em*) genetic background. As shown in Table 2, expression of the *levD* promoter in the *levT*ΔC*/levQ* double mutant was near baseline in all four sugars tested. Since the *levT*ΔC deletion alone led to elevated expression of the *lev* genes in glucose and especially galactose, these results reinforce that LevQ is essential for the function of the LevSR two-component system, whereas the interaction between LevT and LevQ appears to be required for signal perception by the complex.

### Analysis of LevQ by GPS®-LS linker-scanning mutagenesis

**Figure 3:**
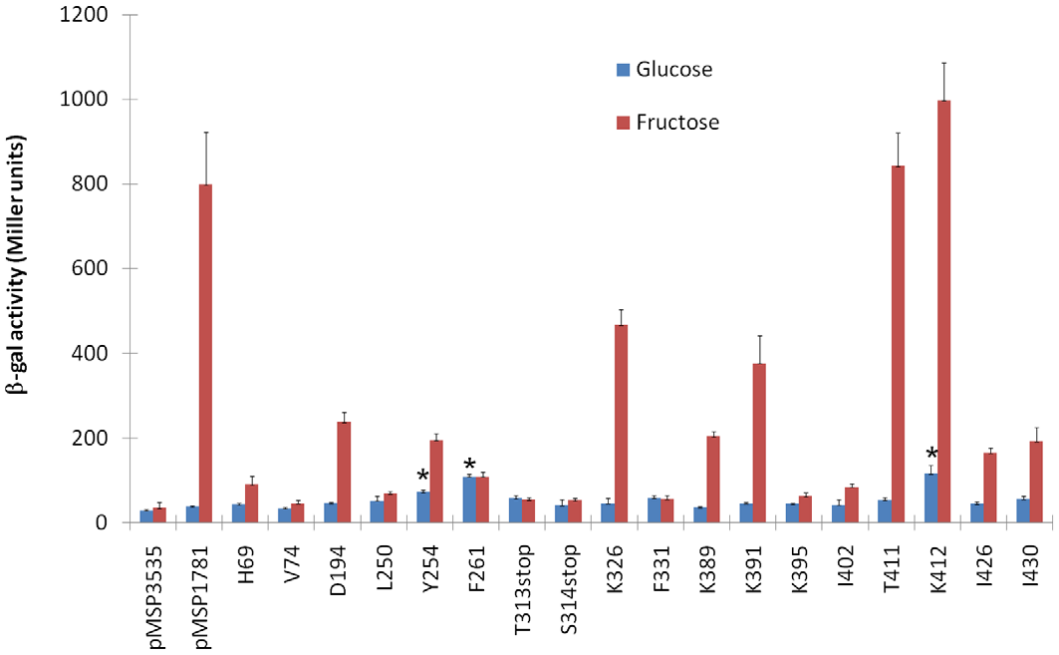
Expression of *fruA* in various *levQ* linker scanning mutants. β-galactosidase activities expressed from various GPS®-LS mutants of *levQ* in cells growing exponentially in TV with 0.5% of glucose or fructose. The results represent the expression levels of a P*fruA*Δcre*-lacZ* fusion (BSCZ) [9]. pMSP3535 - empty vector; pMSP1781 - pMSP3535 expressing *levQ*; others, the sites of insertion of the pentapeptide from the LS cassette (e.g., H69 – a pentapeptide after histidine residue 69) or truncation mutants (e.g., T313stop, a stop codon after threonine 313). Asterisks indicate mutants with significantly higher activities (P value<0.05 by Student's *t*-test) than in the strain containing pMSP1781 when growing in glucose. The results are derived from a minimum of three independent cultures.

In the background of a *levQ* (*sp*) deletion mutant, expression of the P*fruA*Δcre*-lacZ* promoter fusion (BSCZ) [9], which requires LevR for activation but lacks the CcpA binding site (CRE), was reduced to background levels. Expression of the *fruA* promoter could be rescued in this strain by the introduction of pMSP1781, carrying a wild-type copy of *levQ* on plasmid pMSP3535 (Figure 3). Utilizing a commercially acquired GPS®-linker scanning (LS) mutagenesis system, random insertions of 5 amino acids or truncations were introduced into the coding sequence of the plasmid-borne *levQ* gene, generating a library of clones of *levQ* mutants. When introduced into the background of *levQ/*BSCZ, these 18 mutants produced various levels of β-galactosidase activities in response to glucose or fructose (Figure 3). When compared to the positive control strain, *levQ*/BSCZ/pMSP1781, a majority of the mutants exhibited lower expression from the *fruA* promoter when induced by fructose. Insertions in these mutants could be localized to the putative sugar-binding domain and to a smaller region in the C-terminus of LevQ. Interestingly, we also showed that two mutants QLS35 and QLS46, which introduced the pentapeptides VFKHF and CLNNY after amino acid F261 and Y254, respectively, yielded elevated levels of β-galactosidase activities from the *fruA* promoter when growing in non-inducing conditions on glucose (Figure 3). Plasmids harboring these two mutant alleles were also introduced into a *levQ^+^/*BSCZ background in which the *recA* gene had been disrupted via allelic exchange mutagenesis with a *km* cassette to ensure that homologous recombination between the two *levQ* alleles was not responsible for the phenotype. The results showed that even in the presence of a wild-type allele of *levQ*, the QLS35 and QLS46 mutants caused higher levels of *fruA* expression when growing in glucose (data not shown). Additional tests also showed higher expression of the *levD* promoter in these two mutants when cells were growing in glucose or galactose (data not shown). Clearly, insertions into the predicted sugar-binding domain of LevQ alleviated the requirement for normal substrates to be present for activation of the complex.

### Linker-scanning mutagenesis of LevS

**Figure 4:**
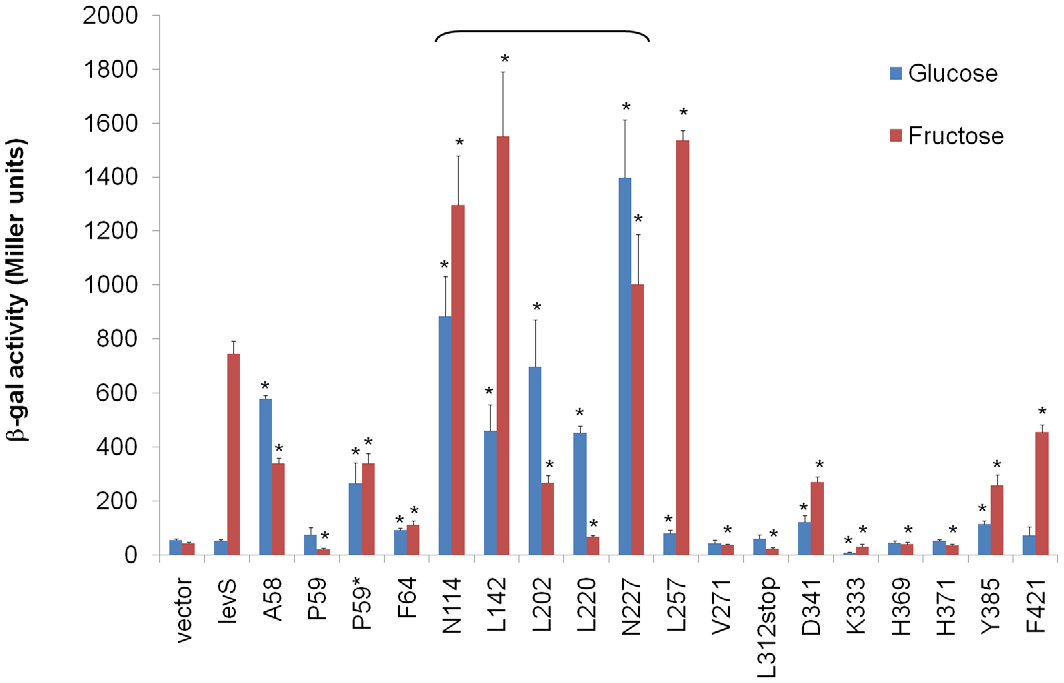
Expression from the *fruA* promoter in strains expressing various *levS* mutant genes. β-Galactosidase activities were measured using various GPS®-LS mutants of *levS* growing exponentially in TV with 0.5% of glucose or fructose. Each result originates from at least three independent cultures and reports the expression levels of a P*fruA*Δcre*-lacZ* fusion (BSCZ). Vector, pMSP3535; *levS*, pMSP3535 carrying wild-type *levS*; others, sites of insertion (P59 and P59* have different insertions) or truncation. Asterisks over the bars indicate activities statistically different (P<0.05 by Student's *t*-test) than those obtained using the strain complemented with a wild-type *levS.* The results are derived from a minimum of three independent cultures.

By applying GPS®-LS linker-scanning mutagenesis to a *levS* fragment carried on plasmid pMSP3535, we also constructed a library of 18 insertion mutants of *levS* in the background of a *levS* deletion strain containing the BSCZ promoter:*lacZ* fusion [9]. These strains were assayed for their β-galactosidase activities while growing on glucose or fructose (Figure 4). Insertions into the C-terminal portion of the histidine-kinase domain mostly caused a loss of expression, regardless of the growth carbohydrate. In contrast, insertions in the N-terminal portion of LevS, in particular the first three transmembrane domains, had less impact on the function of the complex. These findings are consistent with the fact that the kinase domain, located beyond the first 250 amino acids of LevS, is considered essential for the phosphorylation of LevR. Interestingly, in mutants containing insertions within or around the fourth and fifth transmembrane domains of LevS, as indicated by a bracket in Figure 4, aberrant expression from the *fruA* promoter in response to glucose was noted. Among these five variants, the SLS65 (L202) mutant was selected for further analyses. In particular, the same mutation present in SLS65 was reconstructed in single copy in the *S. mutans* chromosome in strain UA159/P*levD-cat* using the PCR-based approach described in the methods sections, resulting in strain SLS65/P*levD-cat.* As detailed in Table 4, the chromosomally-borne variant of SLS65 led to markedly elevated expression of the P*levD-cat* promoter fusion in cells growing in glucose or galactose, whereas expression in fructose or mannose was slightly lower than in the wild-type background. Therefore, the 5-amino acid (VFKHL) insertion into the transmembrane domain (TM5) of LevS may have altered signal perception by the four-component system, resulting in aberrant expression of LevR-regulated genes in non-inducing carbohydrates.

**Table 4:** Expression of *levD* promoter:*cat* fusion in wild type UA159, reconstituted GPS®-LS mutant SLS65 and its derivatives, as measured by CAT assays.

Strain	Avg CAT sp act SD^a^ on various growth carbohydrates			
	Glucose	Fructose	Mannose	Galactose
***levS^+^***	2.8±0.4	474.0±44.7	870.4±85.2	9.6±0.6
***levS*** **LS65**	1,101±34.3	328.3±9.0	580.1±32.0	1,892.2±11.4
***levS*** **LS65/** ***levQ***	1.3±1.8	0.3±0.3	2.7±2.1	10.2±1.9
***levS*** **LS65/** ***levT*** **M1stop**	204.7±4.1	31.0±2.6	61.2±1.9	106.5±11.3

a The data are presented as the average results of at least three independent cultures. Cells were cultured in TV broth with 0.5% of the indicated carbohydrates and assays were performed as described in Materials and Methods. Activity is expressed as nmol of chloramphenicol acetylated (mg of protein^−1^×min^−1^).

In order to probe the role of the sugar-binding components in affecting LevS-dependent perception of signal, the *levQ* or *levT* genes were mutated in the strain carrying the *levS* SLS65 mutation and a P*levD-cat* fusion (SLS65/P*levD-cat*). As presented in Table 4, concurrent deletion of *levQ* in the strain with the SLS65 mutation resulted in nearly complete loss of *levD* expression in all sugars tested. Loss of LevT in SLS65, due to a point mutation (*levT*M1stop), led to uniformly lower, albeit still significant, CAT activities in these conditions. Further tests performed on the other linker scanning mutants containing insertions at L220, A224 and N227 of LevS (Figure 4) in a *levQ* deletion background also indicated that an intact LevQ is required for the phenotype observed in the *levS* mutants (data not shown). Collectively, these results support that the interaction between the histidine kinase LevS and both sugar-binding proteins, LevT and especially LevQ, is a critical factor in the function of the signal transduction complex. Further, transmembrane domains TM4 and TM5 of LevS, and possibly the region between TM5 and the kinase dimerization and phospho-acceptor domain, are particularly important for this interaction.

### LevQ and LevT cysteine-to-alanine mutants

As both LevQ and LevT are required for the function of the LevQRST complex, we began to probe their involvement in potential tertiary structures by replacing their cysteine residues with alanine (see Text S1 for detail). Collectively, our results (Text S1) do not support that there is an absolute requirement for cysteine residues in LevQ or LevT to achieve a tertiary structure that is competent for signaling by LevQRST.

### Linker-scanning mutagenesis of LevT

**Figure 5:**
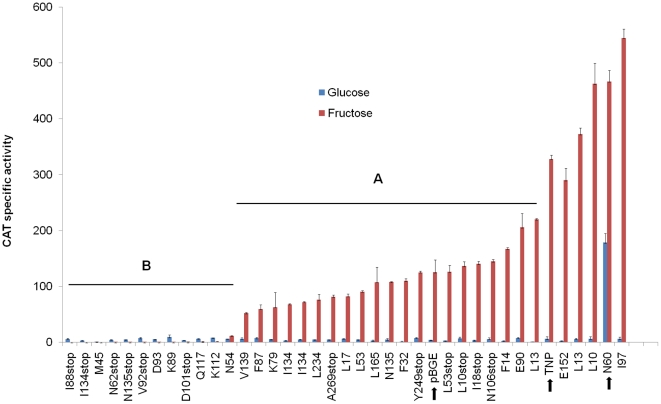
CAT activities of the GPS®-LS mutants of *levT.* The graph shows expression levels of a P*levD-cat* fusion [10] in the background of the *levT*M1stop mutant with various TLS mutants integrated at the *gtfA* site via the pBGE vector (See text for more details). TNP, a promoterless wild-type *levT* sequence expressed from the *gtfA* gene promoter; others, sites of insertion or truncation. CAT spc. activity on the y-axis is nmol of chloramphenicol acetylated (mg of total protein)^−1^ (min^−1^). Activities (when growing in fructose) in group A mutants are within 0.5- to 2-fold that of the strain containing the pBGE vector only, whereas mutants with lower activities are in group B. pBGE, TNP and TLS13 (N60) are highlighted by arrows. The data are from at least three independent cultures growing in TV with 0.5% of glucose or fructose.

Whereas successful complementation of *levQ* or *levS* deletions was achieved by introducing a wild-type *levQ* or *levS* sequence on plasmid pMSP3535, efforts to clone the *levT* gene in *E. coli* in a configuration that would allow for expression were unsuccessful. To circumvent the problem of apparent toxicity of LevT in *E. coli*, a conditional expression vector pBGE [18] was used to clone a promoterless *levT* sequence. The cloning site in pBGE is flanked by two fragments of the *gtfA* gene of *S. mutans*, such that the gene can be integrated into the *gtfA* site and expressed from the native *gtfA* promoter [18]. Introduction of the *levT* construct (pBGE-TNP) into a *levT* mutant (TM1stop) resulted in partial complementation, with P*levD-cat* expression in fructose reaching 70% of that observed in the wild-type background (Figure 5). We believe the partial complementation may be related to the relatively low expression from the *gtfA* promoter under the conditions tested [18], but the expression level was adequate to compare wild-type and mutant variants of *levT* in the same expression system. GPS®-LS mutagenesis was applied to the integration construct, creating a library of 37 *levT* LS mutants (TLS). After transforming strain TM1stop carrying the P*levD-cat* fusion, each mutant was cultured on glucose, fructose or mannose and assayed for CAT activity. As presented in Figure 5, the majority of the TLS mutants (group A, 20 of 37) produced CAT activities comparable to that of the vector control (pBGE), of which six were truncation mutants with translation stops at the 10^th^, 18^th^, 53^rd^, 106^th^, 249^th^ and 269^th^ amino acid residue. Group B mutants, most of which produced lower CAT activities than that of the vector control, contained 12 TLS mutants; six of which had translation stops at the 62^nd^, 88^th^, 92^nd^, 101^st^, 134^th^ and 135^th^ amino acid. One possible interpretation of these results is that the amino-terminal portion of LevT alone, where the transmembrane domain resides, has the ability to interact with other components of the LevQRST pathway and deliver a negative signal. In contrast to the truncation mutants, two TLS mutants expressed CAT activities comparable to those of the wild-type background (TNP), and three TLS mutants gave significantly higher activities than strain TNP. Especially interesting, mutant TLS13, containing an insertion of a VFKQN pentapeptide after Asn60, produced 30-fold higher CAT activity than strain TNP while growing on glucose, and modestly higher expression in fructose and mannose. In fact, when compared to the TNP strain complemented with a wild-type *levT* gene, TLS13 had significantly increased *lev* expression when growing in galactose (8-fold), cellobiose (11-fold), lactose (5-fold), sorbitol (58-fold) or melibiose (9-fold) (Table 3 and data not shown). Concurrent disruption of *levQ* in the background of the TLS13 mutant once again reduced the expression of the P*levD-cat* fusion to near baseline levels (data not shown). These results indicate that LevT, in conjunction with LevQ, has the capacity to modulate the overall activity, and possibly the substrate specificity, of the LevQRST pathway.

## Discussion

The LevQRST four-component regulatory system, composed of a two-component system (LevSR) flanked by two apparent ABC-type sugar-binding proteins (LevTQ), was first identified as a regulator of fructanase A (*fruA*) expression in *S. mutans* [9]. While the classical TCST components of this complex (LevRS) are essential for the expression of the *fruAB* and *levDEFG* operons, LevQ and LevT are also required for activation of these operons by LevSR. Fructose and mannose have been identified as the apparent cognate inducing signals for the complex [10]. While an increasing number of TCST systems are being shown to be regulated by auxiliary factors [8], there is mounting evidence that bacterial solute transporters play essential roles as sensors in a variety of signal transduction and gene regulation pathways [19]. For example, components of the bacterial PTS are well known to participate in a broad range of regulatory functions in *S. mutans* [20]. Recently, members of our laboratory showed that a non-PTS transporter in *S. mutans*, the AguD antiporter of the agmatine deiminase system (AgDS) [21], controls AgDS gene expression by interfering with activation of the operon by the membrane-anchored AguR DNA binding protein in the absence of exogenous agmatine [21]. Interestingly, LevQ and LevT appear to have evolved from ABC-type substrate-binding proteins into sensors that function in concert with the LevRS TCST couple. Importantly, no functional ABC transporters are encoded near the *levTSRQ* operon and the cognate substrates for the LevQRST system, fructose or mannose, appear to be transported exclusively by the PTS and not by ABC transport systems [9], [18], [22]. Therefore, the only function of LevQ and LevT seems to be their role as part of the LevQRST signaling complex.

Based on results presented in this report, we propose the following models regarding the individual functions of, and interactions between, the members of LevQRST. First, LevQ is required for determining substrate specificity, most likely by sensing the presence of specific extracellular carbohydrates. Multiple lines of evidence support this role, including that a *levQ*C161A mutation produced increased *levD* expression in the presence of glucose, fructose and galactose, but decreased expression on mannose. Likewise, the *levQ*con mutations (*levQ*E170A and *levQ*F292S) caused higher levels of *levD* expression on glucose, galactose, sorbitol, melibiose or cellobiose, but little expression in the presence of fructose, mannose or sucrose. Interestingly, compared with the phenotype of a *levT* null mutant (*levT*M1stop), the *levQ*con/*levT*M1stop double mutant showed a similar change in *levD* expression as the LevQcon strain. Thus, the *levQ*E170A/F292S mutations altered the specificity of the signaling complex independently of LevT. On the other hand, deletions of *levQ* almost always led to drastic reductions in the activity of the *fruA/levD* promoters, similar to levels observed following deletion of *levR*. These effects were seen whether the *levQ* deletions were assessed in the otherwise wild-type background or when they were introduced concurrently into the background of *levT*M1stop, *levT*ΔC, *levS*LS65 or *levT*LS13. Thus, it seems that LevQ is also required for the overall functionality of this complex.

The data also provide evidence that the function of LevT may be less critical than that of LevQ, and that LevT may contribute more to the proper sensing of substrates than activation of the sensor kinase. First, if *levQ* is deleted *levD* promoter activity is lost, whereas loss of *levT* reduced *levD* promoter activity and the extent of reduction was dependent on the carbohydrate source. Specifically, expression levels of LevR-regulated genes was comparable to that in the wild-type background in glucose, was significantly higher in galactose, and was greatly reduced in cells growing in fructose or mannose. Notably, a much greater reduction in *fruA/levD* expression was seen when the N-terminal transmembrane domain of LevT was kept intact, indicative of the ability of this region to deliver a negative signal to LevSR, even in the absence of its sugar-binding domain. In support of this model, 6 LevT-LS mutants with significant C-terminal truncations, but intact N-terminal transmembrane domains, produced lower P*levD-cat* expression than those seen with the empty vector control (Figure 5). Thus, LevT is critical for substrate selectivity and operates centrally in regulating the signal transduction system. Somewhat similar to the LevQRST system is an essential TCST system required for cell wall maintenance in *Bacillus subtilis*, YycGF, where the auxiliary regulators YycH and YycI are believed to interact with the sensor kinase YycG via their transmembrane domains to negatively regulate the function signal transduction system [23]. However, results obtained here clearly indicate that LevQT function beyond simply negatively modulating the activity of the sensor kinase and response regulator.

As presented in this report, we had some initial success with the strategy of cross-linking coupled with Western blotting to show that LevQ was exposed on the cell surface. However, the inability to detect LevT or LevS signals in Western blots using antisera generated against recombinant LevT or LevS fragments has hindered progress toward biochemical detection of interactions between LevQ, -S and -T. As noted, it appears this problem is due to the combination of very low levels of production of these proteins, a lack of stability of the proteins, and the quality of the antisera. Notwithstanding, since deletion of *levS* led to complete loss of *levD/fruA* operon expression [9], the altered expression of LevR-controlled genes in some of the *levS* mutants in this study cannot be attributed to instability of the mutant proteins. This is also the case for the LevQ and LevT variant proteins (Table 1, 2; Figure 3, 5).

As reported previously by us, expression of the *fruA* and *levD* operons is subject to carbon catabolite repression (CCR), both with and without the direct involvement of CcpA [10], [14]. Although LevR is required for CcpA-independent CCR, a process apparently involving seryl-phosphorylated HPr and the EII^AB^ (ManL) component of the EII^Man^ permease, our data do not exclude the possibility that CCR of *fruA/levD* may also be influenced by the sugar-binding proteins LevQT [10]. In fact, it is possible that, for some of the LevQT mutants, altered expression from the *fruA/levD* promoters occurred as a result of CCR mediated through LevQRST rather than a change in substrate specificity of the mutants. To test this possibility, three of the mutants constructed in this study (*levQ*con, *levT*C149A and *levQ*C161A) were evaluated in a strain that also carried a deletion of the *manL* gene, a mutation that results in dramatic alleviation of CCR of the *fruA/levD* operons [10]. The resultant strains showed generally increased expression of the P*levD-cat* fusion due to the loss of ManL when growing on all sugars tested (data not shown), suggesting that the effects of the *levQ* and *levT* mutations are independent of ManL-dependent CCR. While it is beyond the scope of the present study to test CCR effects in other LevQT mutants, the experiments performed to date add support to our current working model in which LevR is the primary target in the LevQRST system for CCR of the *fruA/levD* operons [14].

Finally, despite the fact that LevSR have classical characteristics of conventional sensor kinases and response regulators, LevSR are clearly unable to function in the absence of the sugar-binding proteins. While LevR does function as a typical response regulator [9], computer analysis suggests that only limited portions of the LevS protein are exposed to the extracellular environment, which seems to be common for TCST systems with auxiliary components [8]. Instead, significant roles for LevQ and LevT in signal sensing are evident, and these proteins may in turn transduce the carbohydrate signals to LevS, perhaps through interactions between transmembrane domains. Such a model is best supported by the isolation of 5 GPS®-LS mutants (including *levS*LS65) with insertions concentrated around the transmembrane domains TM4 and TM5 (Figure 4). All of these mutants showed aberrant expression of the P*levD-cat* fusion in the presence of glucose, and this phenotype required the presence of an intact LevQ protein. Notwithstanding, the fact that loss of *levT* in the background of *levS*LS65 also significantly reduced the overall expression from the *levD* promoter provides support that LevT also participates in the signal transduction process. Further experimentation has been planned to study protein-protein interactions directly once obstacles related to expression levels and sensitivity of the immunoblotting can be overcome.

In conclusion, this study begins to dissect the roles in sensing and signaling of the components of a complex and unusual bacterial signal transduction system required for expression of a known virulence attribute of a human pathogen. Given the levels of sequence conservation observed between the LevQRST operon in *S. mutans* and its homologues found in several other important Gram-positive bacteria, we expect these systems to have a reasonably high degree of conservation in mechanisms of signal transduction and gene regulation. Moreover, as additional TCST systems with secondary regulators are disclosed by bacterial genome sequencing and functional studies, knowledge regarding the function and structure relationships of the LevQRST complex should prove valuable for expanding our understanding of the interactions between core TCST components and accessory regulators. Also, given the established role of fructan metabolism by FruA in virulence [6] and the critical role LevDEFG play in carbohydrate transport and gene expression [9], [10], further analysis of the mechanisms of control by LevQRST could lead to novel therapeutics to compromise the virulence of an important human pathogen [24].

## Materials and Methods

### Bacterial strains and growth conditions

*S. mutans* strain UA159 and its derivatives were grown in brain heart infusion media (Difco Laboratories, Detroit. MI) at 37°C in a 5% CO_2_ - 95% air atmosphere. *Escherichia coli* strain DH10B was maintained in Luria-Bertani medium at 37°C in air. Antibiotics were used when necessary at the following concentrations (µg/ml^−1^): for *S. mutans,* kanamycin (Km) 500 (in liquid media) or 1000 (in agar plates), erythromycin (Em) 5 or 10 and spectinomycin (Sp) 500 or 1000; for *E. coli*, Km 25, Em 300 and Sp 50. For Chloramphenicol acetyltransferase (CAT) and β-galactosidase assays, *S. mutans* strains were grown in tryptone-vitamin (TV) base medium [11] with the specified concentrations of carbohydrates.

### DNA manipulation

Standard techniques [25] were employed to create recombinant DNA fragments and plasmids. All restriction and modifying enzymes were purchased from New England Biolabs (Beverly, MA) and used according to protocols provided by the supplier. Primers for PCR amplifications were synthesized by Integrated DNA Technologies, Inc. (Coralville, IA).

### Engineering of nuclease-fusion strains

To assess whether the components of the signal transduction pathway could be surface-localized, a signal-peptide-free staphylococcal nuclease sequence (Δ_SP_Nuc)[15] was fused to LevT, LevQ and LevS using a modified ligation-transformation strategy [26] (illustrated in Figure S4). To create the LevT-Δ_SP_Nuc fusion, primers 1784(ABC)-55 (5′- ATG GTA GTA AGG GAA GTC TCA TCT C -3′) and 1784(ABC)-53-RI (5′- TCG AAT TCT TTC TTG AGC ACA CAG TAC -3′) were used to amplify a 1-kbp DNA fragment containing *levT* and some of its 5′ flanking sequence. This DNA fragment was subsequently digested with *Bam*HI, targeting a unique *Bam*HI site ∼200 bp from the N-terminus of the *levT* coding sequence, releasing fragment A. Another DNA fragment containing the downstream *levS* sequence was also generated using primers LevS-Nuc-5′ (5′- GGG AAG GAT CCT TTA ACA GGG TGG CAG T -3′) and LevS-Nuc-3′ (5′- GCC CCA AGG GAT CCT GAA TTT CTC T -3′), which was then digested with *Dra*I to release fragment B. Meanwhile, a 2.3-kbp DNA fragment carrying Δ_SP_Nuc followed by an erythromycin resistance marker (*em*) was released via digestion with *Bam*HI and *Hpa*I from plasmid pFUN [15], a gift provided by Dr. Isabelle Poquet. The Nuc fragment was then ligated with fragment A and B to allow in-frame fusion of the N-terminal *levT* sequence with Δ_SP_Nuc and insertion of the *em* marker between the *levT* and *levS* sequences. Homologous recombination between the *levTS* sequence in the chromosome and the ligation product ensures insertion of the *em* marker and the simultaneous addition of Δ_SP_Nuc to the C-terminus of the *levT* gene. Fusions of LevS and LevQ to Δ_SP_Nuc were created by the same strategy, with Δ_SP_Nuc fused behind the 253^rd^ amino acid of LevS and the 193^rd^ amino acid of LevQ, respectively. All strains were confirmed by PCR and DNA sequencing.

### GPS®-LS linker-scanning mutagenesis

Mutagenesis of *levQ, levT* and *levS* sequences was performed with the GPS®-LS linker-scanning (LS) system (New England Biolabs) according to the supplier's instructions and protocols described elsewhere [21]. The GPS®-LS system allows for random insertion of 15 nucleotides into the gene of interest, with four of the six possible reading frames creating a five-amino-acid insertion, and the other two creating stop codons. A nisin-controlled expression vector pMSP3535 [27] was used to clone the *levQ* and *levS* sequences, resulting in plasmids pMSP1781 and pMSP1783, respectively. No nisin was added to the culture medium for the purpose of inducing the expression of these inserted sequences, since the basal level of expression from the *nisA* promoter was sufficient for complementation of *levQ* and *levS* mutants. However, multiple attempts to clone *levT* into pMSP3535, with or without its native promoter sequence, produced no viable clones, suggesting that the gene product of *levT* was toxic to the *E. coli* host. To circumvent this problem, an integration vector pBGE [18] was used to successfully clone only the ribosomal binding site (RBS) and the coding sequence of the *levT* gene, creating plasmid pBGE-TNP. Subsequent integration of the promoterless *levT* into the chromosome within the *gtfA* gene allowed for stable maintenance of *levT* in *S. mutans* in a single copy, such that the expression of *levT* was driven by the native promoter of *gtfA* [18].

GPS®-LS mutagenesis was applied to plasmids pMSP1781, pMSP1783 and pBGE-TNP, each yielding a library of random insertion mutants (Table S1). Selected mutant genes were then introduced into the strains lacking the intact copy of the corresponding gene [9] and the impact of the various LevQST derivatives on the ability of the complex to activate transcription of the *fruA/levD* genes was assessed using the *fruA* or *levD* promoter-reporter gene fusions P*fruA*Δcre-*lacZ* (BSCZ) [9] or P*levD-cat* [10].

### Construction of other mutants

Various mutants were constructed using allelic exchange with non-polar elements encoding resistance to kanamycin (*km*), erythromycin (*em*) or spectinomycin (*sp*) to replace the genes of interest without disrupting downstream gene expression, as detailed elsewhere [18], [26]. In addition, a PCR-based site-directed mutagenesis strategy, reported previously by our group [14], has been improved and was used to create markerless point mutations in the *S. mutans* genome. Briefly, a mutator DNA fragment was created by recombinant PCR to engineer specific changes in the sequence of the target gene, followed by transformation of UA159 using this DNA in combination with a pSU20Erm-based [28] suicide plasmid encoding resistance to Em and a 100-bp internal fragment of the phospho-β-galactosidase (*lacG*) sequence [29]. Competent cells that take up both DNA molecules lose the ability to grow on lactose while acquiring the desired mutation. Em-resistant, lactose-negative transformants were screened using an allele-specific PCR protocol (MAMA PCR- *m*ismatch *a*mplification *m*utation *a*nalysis)[14], [30] for the presence of desired mutations in the chromosome (see Table S2 for allele-specific MAMA primers used in this study). After confirming the mutations by sequencing, the resultant strains were patched onto TV agar containing 0.5% lactose as the sole carbohydrate to identify Em-sensitive revertants that had lost the suicide plasmid due to spontaneous excision. Mutants constructed in this fashion include: strain LevTM1stop, which has the first codon (ATG) of LevT replaced by a stop codon (TAG); strains *levQ*E170A and *levQ*F292S; strains LevTC12A, LevTC149A, LevQC161A, LevQC188A, LevQC296A and LevQC336A, which had the cysteine residues in LevT or LevQ replaced by alanines; and strains LevQLS35/P*levD-cat*, LevQLS46/P*levD-cat* and LevSLS65/P*levD-cat*, which are linker-scanning mutants reconstituted by mobilizing the insertion onto the chromosome.

Strain LevQcon, containing mutations in the *levQ* gene that resulted in constitutive expression of the *fruA* and *lev* operons, was isolated following transformation of strain UA159 with a *levQR*-containing DNA fragment amplified by error-prone PCR [31], along with a small amount (100-fold less than the PCR product) of plasmid DNA carrying the P*levD-cat* fusion and a kanamycin marker [10]. The nature of the mutation was disclosed by sequencing of PCR products obtained from the mutant.

To construct the *levQRST*-overexpressing strain T/ldh, a recombinant PCR reaction was performed to fuse the *ldh* (lactate dehydrogenase) promoter behind a DNA fragment that contains the sequence upstream to the *levT* promoter, using a set of primers *ssbA*-1 (5′- GGC AGG ATT TAA AGC ATATGA ATT AGC -3′), *ssbA/ldh*-FWD (5′- GAG GGG CGT TTG CCA GGA AGC TGG AAG AGC CCG AGC AAC -3′), *ssbA/ldh*-RVS (5′- GTT GCT CGG GCT CTT CCA GCT TCC TGG CAA ACG CCC CTC -3′) and *ldh*-2RI (5′- GTT GCA GTC GAA TTC TAA ACA TCT CCT T -3′). This PCR product, a *km* marker and a DNA fragment containing the complete coding sequence of *levQ* (including the ribosomal-binding site), that was generated using primers *levT*-1RI (5′- GAT AAA AGA ATT CGG AGG AAG TAA TGA AA -3′) and *levT*-2 (5′- GGA TTA GTT GGT AAT TTT TCA CCT TTT AC -3′), were then restriction-digested and ligated together with *km* in between. This ligation product was used to transform strain UA159 and Km-resistant clones were confirmed by PCR and sequencing.

### Cross-linking, cell fractionation and Western blotting

Cells from a 50-ml culture of exponentially growing *S. mutans* were harvested by centrifugation, washed three times with cold PBS (pH 8.0), resuspended in 1 ml of PBS, then treated with the cross-linking reagent bis[sulfosuccinimidyl] suberate (BS^3^) (Thermo Scientific, Waltham, MA) at 3.5 mM concentration at 4°C for 1 h. Reactions were terminated by adding 20 µl of 1 M Tris-Cl (pH 8.0) and incubating at room temperature for 15 min. Cells were washed once with PBS, homogenized in sodium dodecyl sulfate (SDS) boiling buffer (60 mM Tris pH 6.8, 10% glycerol, and 5% SDS) with glass beads, and then centrifuged at 16,000×*g* for 10 min at 4°C. Proteins in the soluble fraction were then subjected to SDS polyacrylamide gel electrophoresis (SDS-PAGE) and Western blot analysis [25].

Cell fractionation was carried out according to a protocol previously developed for *S. mutans* [32] with the following modifications. Briefly, an exponentially-growing bacterial culture (50 ml) was harvested, washed once in 20 ml TE (10 mM Tris-Cl, 1 mM EDTA, pH 8.0) and resuspended in 1 ml TES buffer (50 mM Tris-Cl pH 8.0, 1 mM EDTA and 20% sucrose) that also contained 10 mg/ml of lysozyme, 150 units/ml of mutanolysin and 1 mM PMSF (phenylmethylsulfonyl fluoride). After incubation for 3 h at 37°C with gentle agitation, the cell suspense was centrifuged for 5 min at 3,200×*g* at 4°C and the supernatant fluid was collected as the cell-wall-associated proteins. The cell pellet was then washed three times with 1.5 ml TES buffer before being resuspended in 1 ml of osmotic lysis buffer (50 mM Tris-Cl pH 7.5, 10 mM MgSO_4_, 0.8 M NaCl). Then, 5 µl of 10 mg/ml RNase A, 5 µl of 10 mg/ml DNase I and 50 µl of bacterial protease inhibitor cocktail (Sigma) were added and the mixture was incubated for 30 min at 37°C to ensure complete lysis and degradation of nucleic acid. The bacterial lysis mixture was then centrifuged for 10 min at 14,000 rpm at 4°C using a bench-top centrifuge to pellet the intact cells and debris. The supernate was further centrifuged at 100,000×*g* at 4°C for 1 h to precipitate the cell membranes and the supernatant was kept as the cytoplasm fraction. The pellet was rinsed three times with osmotic lysis buffer and resuspended in 200 µl of protein loading buffer. Both cell-wall-associated proteins and the cytoplasmic proteins were TCA precipitated before being resuspended in 200 µl of protein loading buffer. All protein samples were boiled for 10 min before Western blot analysis.

Recombinant fragments of the LevT, LevQ and LevS proteins were engineered using a vector pQE30 (Qiagen, Valencia, CA) by in-frame fusion of an N-terminal 6×His-tag to the putative sugar-binding domains of LevT (beginning with Thr41) and LevQ (starting with Gly40), or to the C-terminal histidine kinase domain of LevS (at Asn214), respectively. The proteins were then over-expressed in an *E. coli* host by induction with isopropyl-D-thiogalactopyranoside (IPTG) and purified using a Ni^2+^ affinity column as recommended by the supplier (Qiagen). Rabbit antisera were raised against each recombinant protein by Lampire Biological Laboratories, Inc. (Pipersville, PA). Anti-LevQ antiserum was affinity purified against immobilized LevQ antigen before use in immuno-blotting. Western blot analysis was carried out using a horse radish peroxidase-based SuperSignal West Pico Chemiluminescence kit (Thermo Scientific).

### Enzymatic assays

CAT [33] and β-galactosidase [34] assays were performed according to previously published protocols [21]. For nuclease assays, bacterial cells were grown to late exponential phase (OD_600_ ≅ 0.6) in BHI, followed by centrifugation at 14,000×*g* at 4°C for 1 min. The supernatant fluid was transferred to another tube and kept on ice until assays were performed. The cell pellets were washed twice with cold PBS, resuspended in the same volume of fresh BHI medium and used for nuclease assays. The reaction was composed of 100 ng of plasmid DNA of pTZ18R, 1.5 µl of 10× buffer (175 mM HEPES pH 7.5, 275 mM MgCl_2_ and 275 mM CaCl_2_) and 12.5 µl of culture supernate or cell suspension. After incubation at 37°C for 1 h, 10 µl of each reaction was resolved by agarose gel electrophoresis. Controls included fresh BHI medium, cultures from *Staphylococcus aureus*, *S. mutans* UA159 or *S. mutans* UA159 containing the plasmids pVE8009 (positive control) or pVE8010 (negative control) [15].

## Supporting Information

Figure S1 Computer prediction of LevQ localization (http://bp.nuap.nagoya-u.ac.jp/sosui/). Indicated are four cysteine residues (161, 188, 296, 336), Glu170 and Phe292.
(TIF)

Figure S2 Computer prediction of LevT localization. Circled are Cys12 and Cys149.
(TIF)

Figure S3 Computer prediction of LevS structure and localization. Circled are Leu 202, Leu 220, Ala224 and Asn227.
(TIF)

Figure S4 Construction of Δ_SP_Nuc fusions.
(TIF)

Figure S5 LevQ Western blot of various fractions of strain UA159 and *levQ* mutant.
(TIF)

Figure S6 SDS-PAGE using recombinant His-LevQSB protein.
(TIF)

Figure S7 Growth curves of strain UA159, LevQcon and LevTM1stop. (A) 10 mM glucose, (B) 10 mM fructose and (C) combination of fructose (0.05%) and inulin (0.5%).
(TIF)

Table S1 GPS linker-scanning mutants used in this study.
(XLS)

Table S2 MAMA primers used in this study.
(XLS)

Text S1 Phenotype of the cysteine-to-alanine mutants of LevQ and LevT.
(DOC)
