# Supplementary figures and images for: Genetic Analysis of the Functions and Interactions of Components of the LevQRST Signal Transduction Complex of Streptococcus mutans
Source: PLoS One. 2011 Feb 22;6(2):e17335. doi: 10.1371/journal.pone.0017335 (PMC3043104; doi:10.1371/journal.pone.0017335)

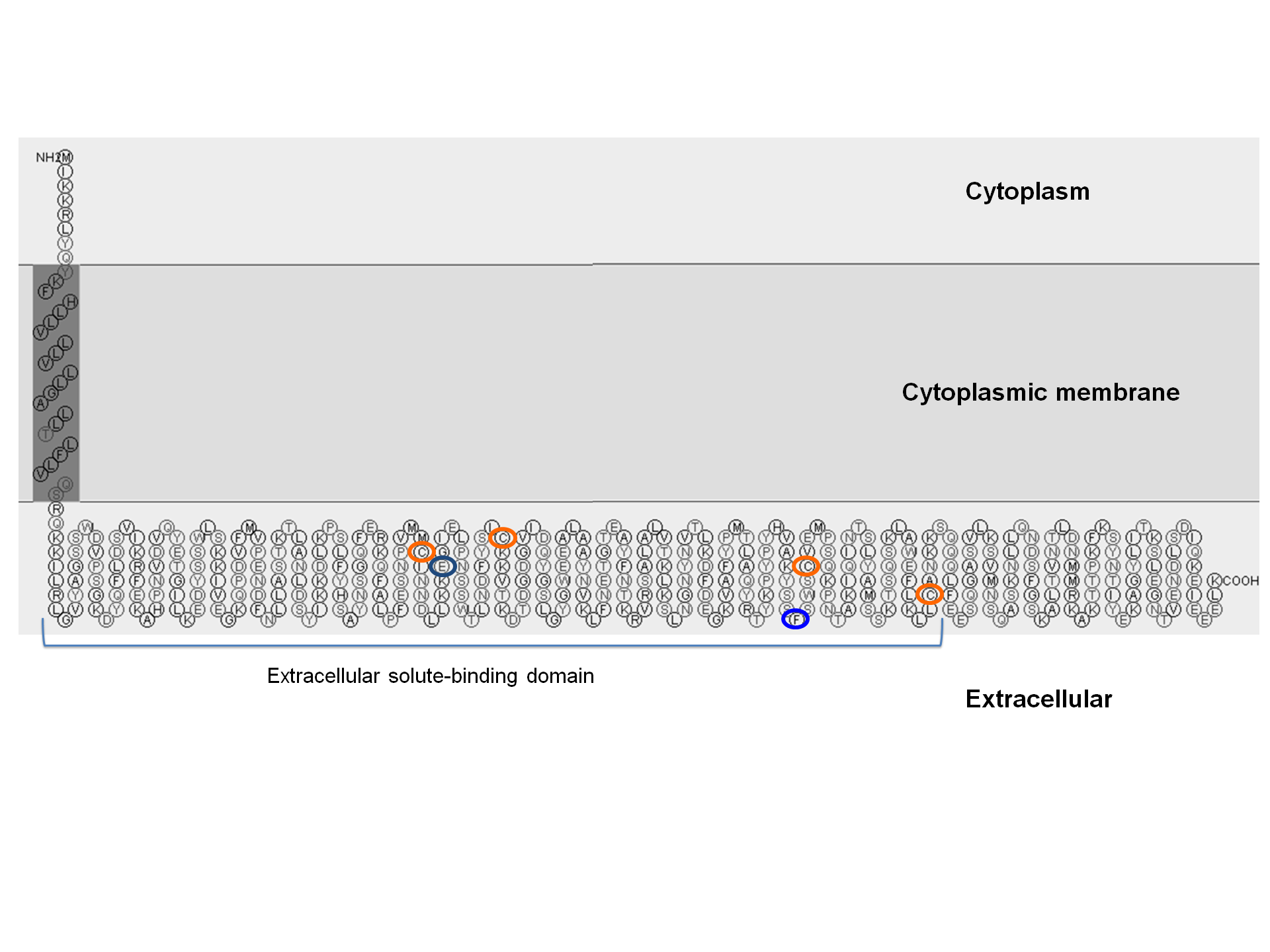

Supplement: Figure S1 — Computer prediction of LevQ localization (http://bp.nuap.nagoya-u.ac.jp/sosui/). Indicated are four cysteine residues (161, 188, 296, 336), Glu170 and Phe292. (TIF) [file pone.0017335.s001.tif]

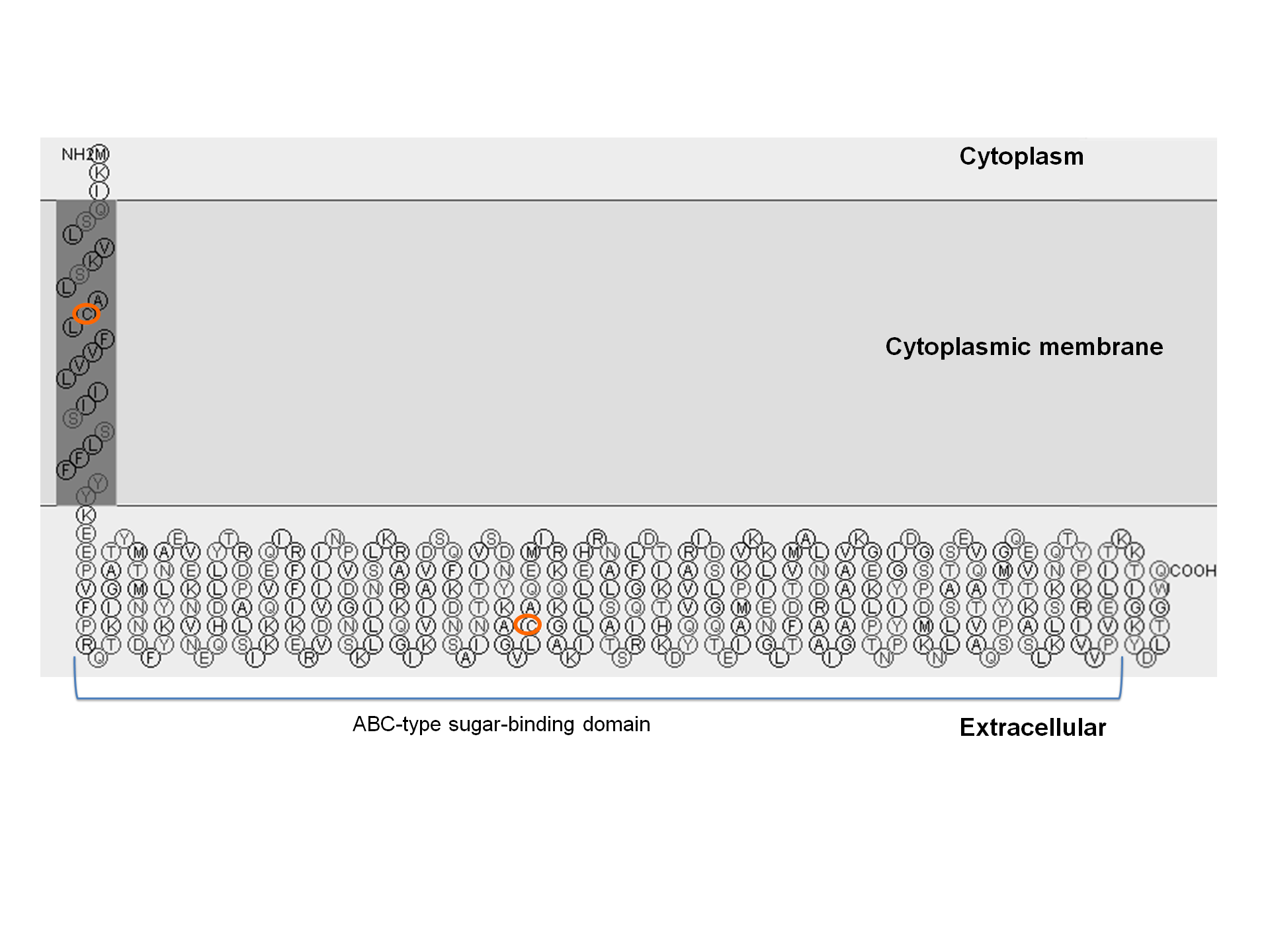

Supplement: Figure S2 — Computer prediction of LevT localization. Circled are Cys12 and Cys149. (TIF) [file pone.0017335.s002.tif]

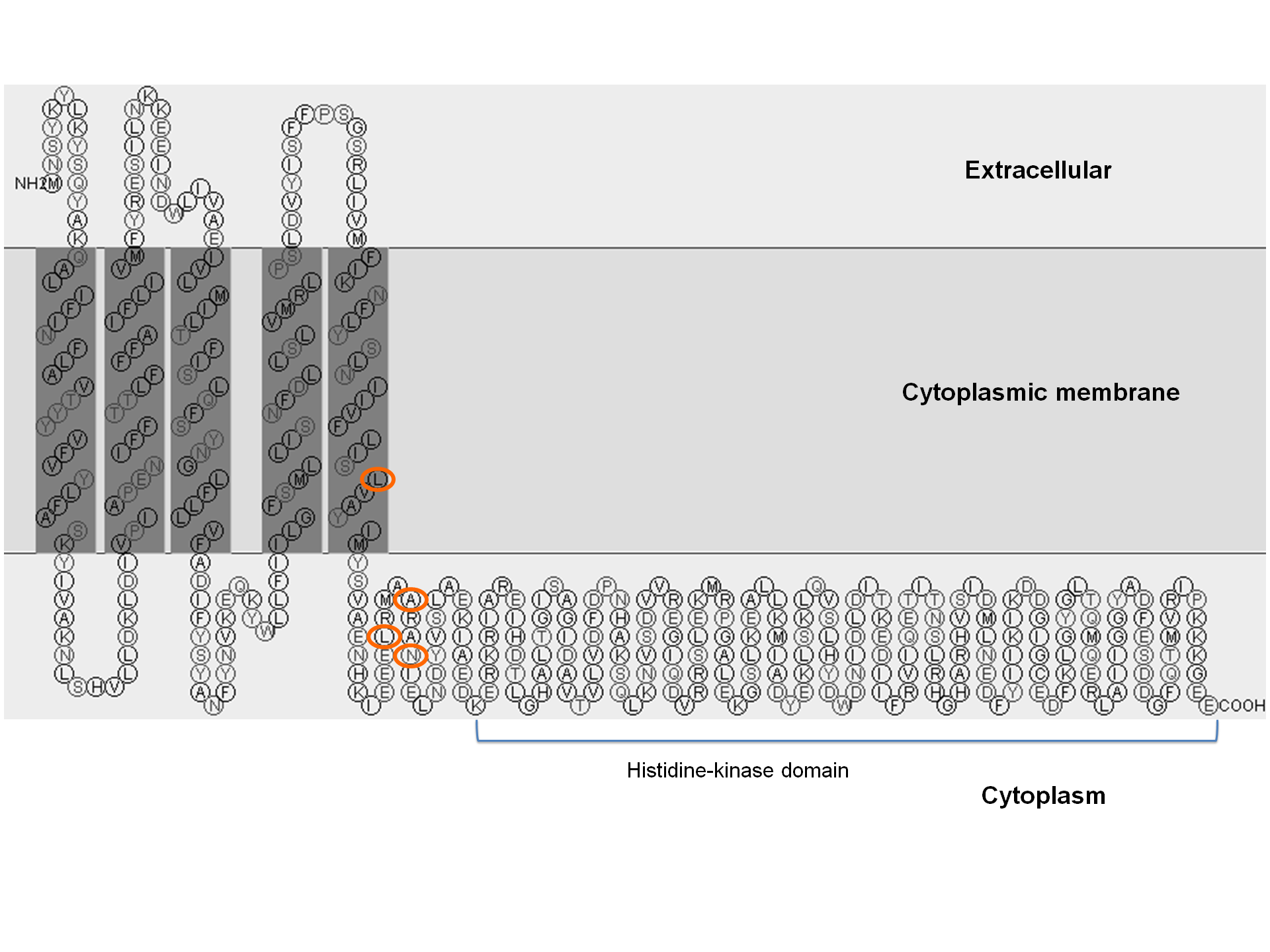

Supplement: Figure S3 — Computer prediction of LevS structure and localization. Circled are Leu 202, Leu 220, Ala224 and Asn227. (TIF) [file pone.0017335.s003.tif]

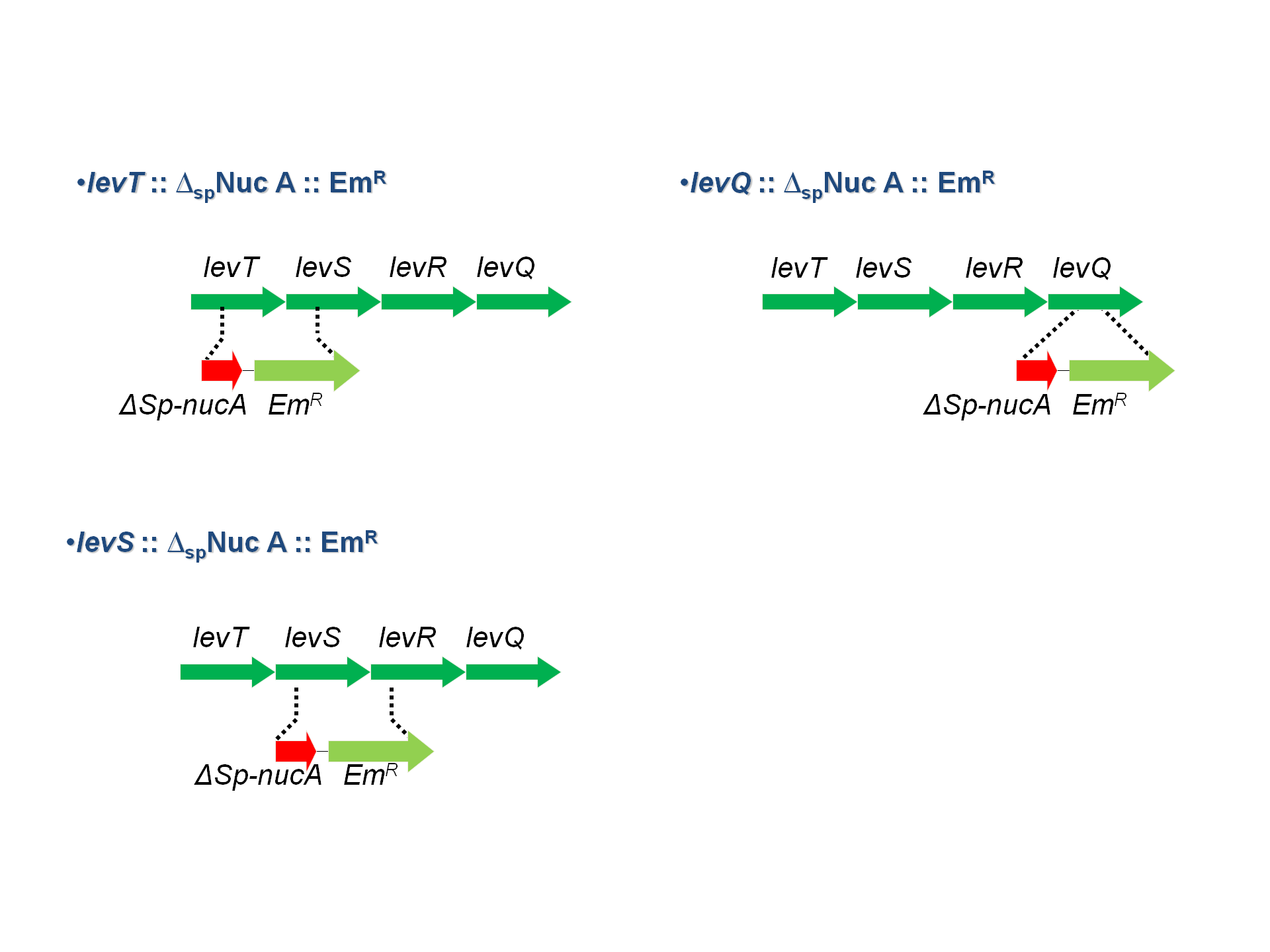

Supplement: Figure S4 — Construction of ΔSPNuc fusions. (TIF) [file pone.0017335.s004.tif]

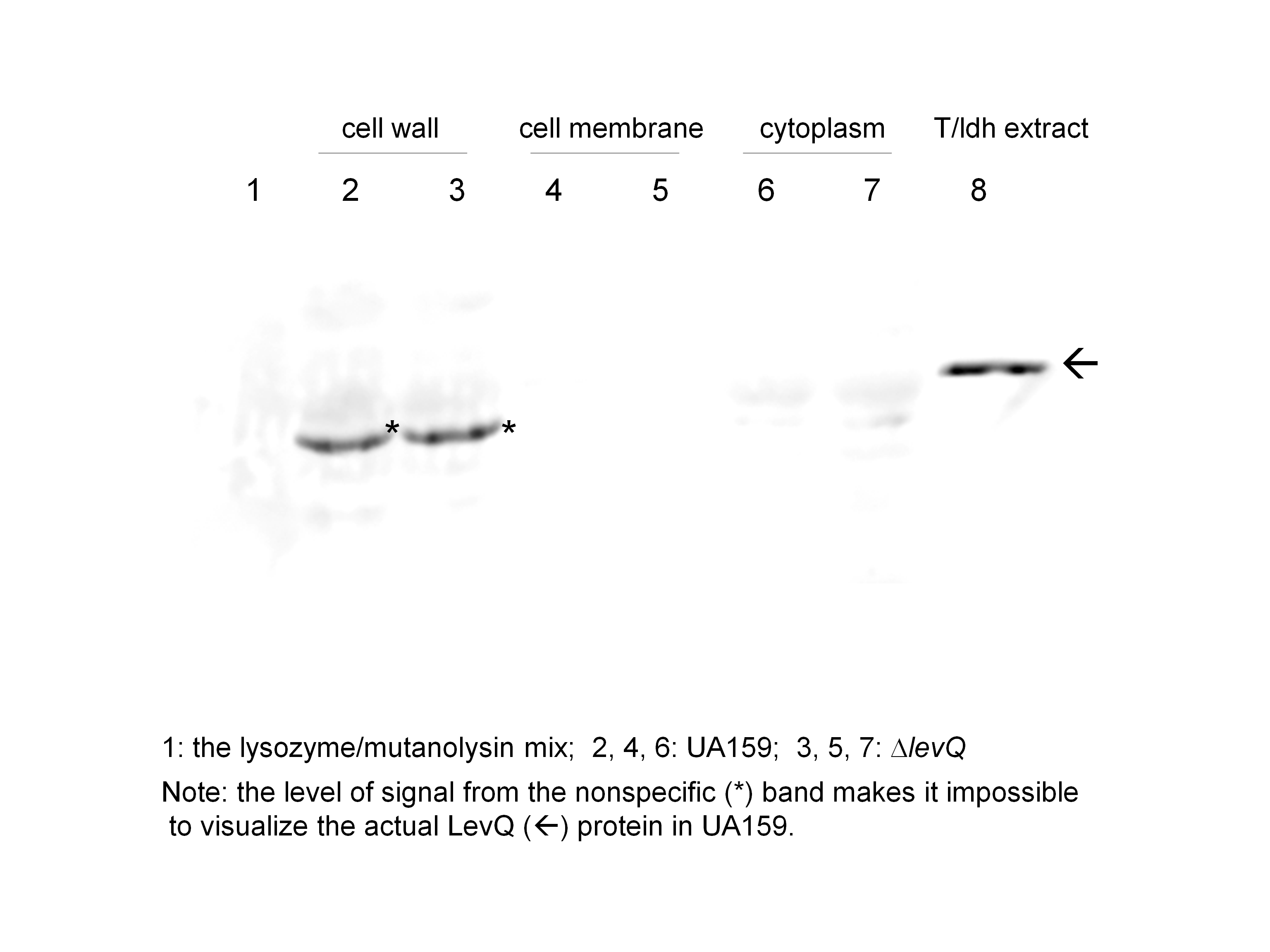

Supplement: Figure S5 — LevQ Western blot of various fractions of strain UA159 and levQ mutant. (TIF) [file pone.0017335.s005.tif]

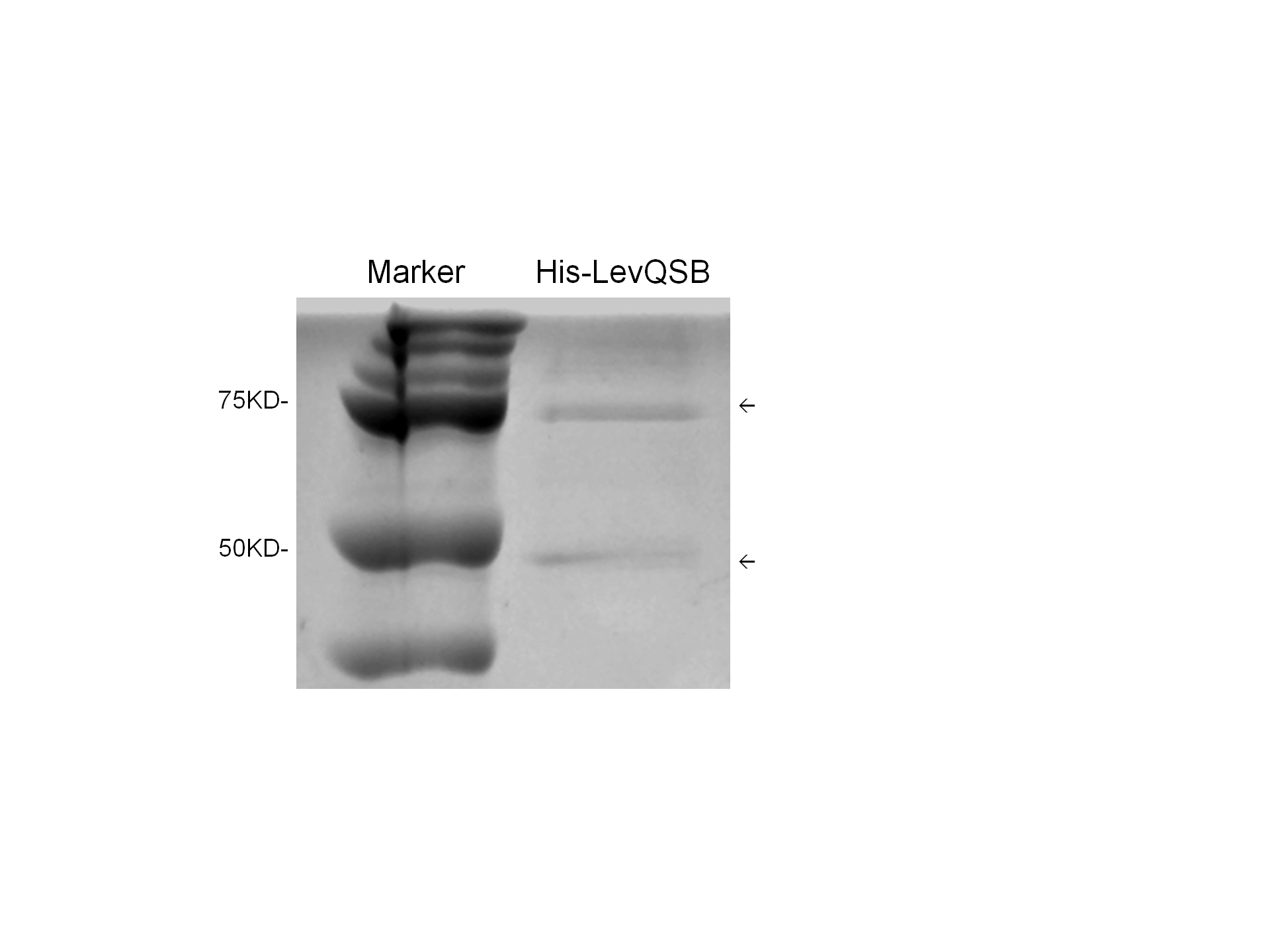

Supplement: Figure S6 — SDS-PAGE using recombinant His-LevQSB protein. (TIF) [file pone.0017335.s006.tif]

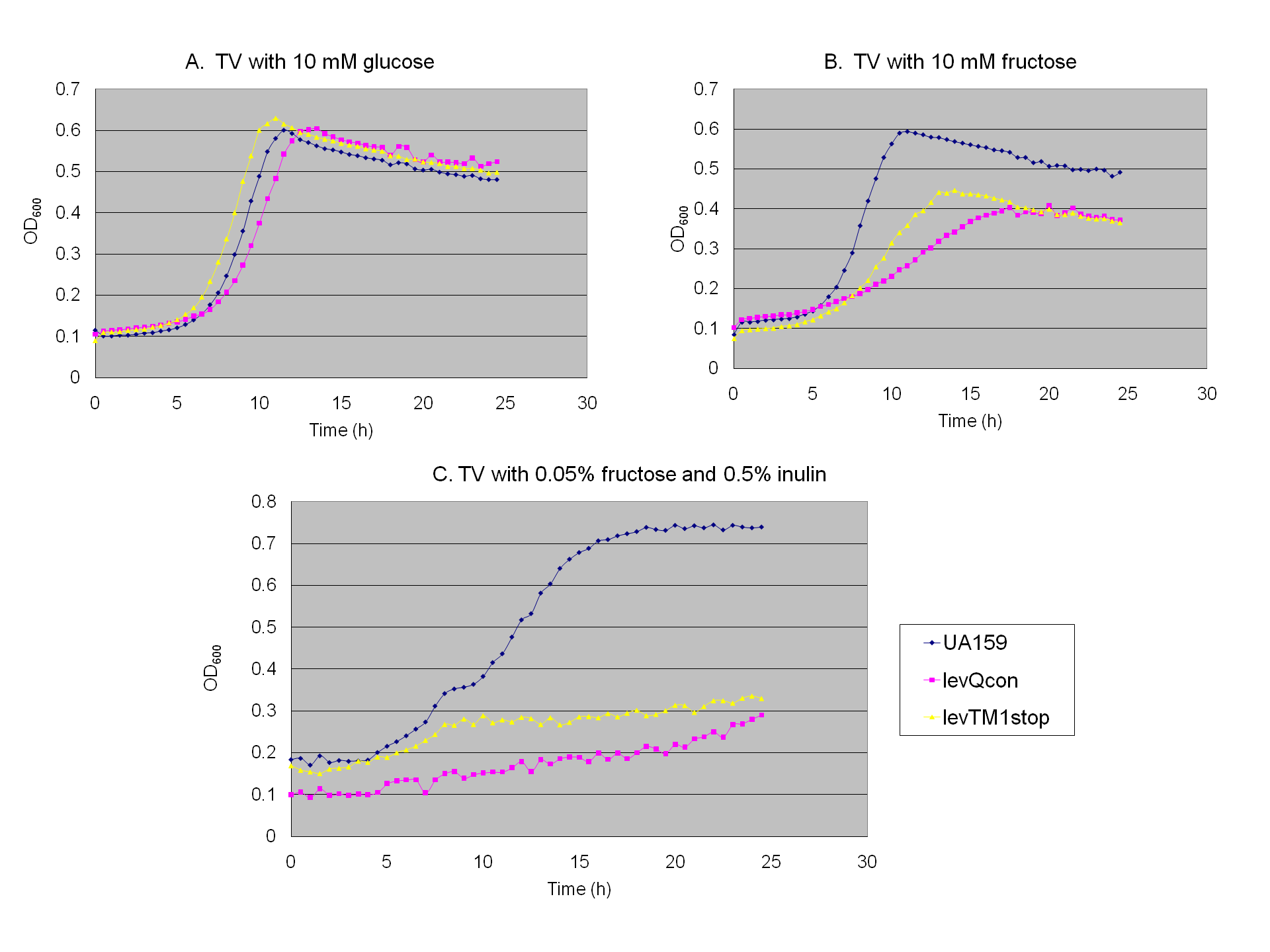

Supplement: Figure S7 — Growth curves of strain UA159, LevQcon and LevTM1stop. (A) 10 mM glucose, (B) 10 mM fructose and (C) combination of fructose (0.05%) and inulin (0.5%). (TIF) [file pone.0017335.s007.tif]
