# Supplementary material for: Genetic Analysis of the Functions and Interactions of Components of the LevQRST Signal Transduction Complex of Streptococcus mutans
Source: PLoS One. 2011 Feb 22;6(2):e17335. doi: 10.1371/journal.pone.0017335 (PMC3043104; doi:10.1371/journal.pone.0017335)
Supplement: Text S1 — Phenotype of the cysteine-to-alanine mutants of LevQ and LevT. (DOC) [file pone.0017335.s010.doc]

**Cysteine-to-alanine mutants of LevQ and LevT**

Four cysteine residues within the LevQ sequence, Cys161, 188, 296 and 336, were replaced individually with alanines via a PCR-based mutagenesis strategy and the impact of each individual mutation was assessed by monitoring the expression levels of the P*fruA*Δcre*-lacZ* (BSCZ) or P*levD-cat* promoter fusion in these mutants. Mutant LevQC161A gave the most significant change in *levD* promoter activity compared to the wild-type genetic background (Table S3; BSCZ data not shown), with 20- and 40-fold higher CAT activities in galactose and glucose, respectively. CAT activities in fructose cultures of LevQC161A were modestly higher than those in the wild-type background, but mannose-grown cells showed slightly lower activities. However, the other three cysteine-to-alanine mutants, LevQC188A, LevQC296A and LevQC336A, presented little change in the levels of *fruA/levD* gene expression under all conditions tested.

Computer algorithms identified a putative transmembrane segment in the LevT sequence near the N-terminus, with a cysteine residue (Cys12) located in the center of this domain. A homologous protein from *B. subtilis,* RbsB, has been reported to be a membrane-anchored extracellular lipoprotein responsible for high-affinity D-ribose transport via an ABC transport system [1]. MsmE in *S. mutans,* a substrate-binding component of the multiple sugar metabolism (*msm*) [2] is also anchored to the cell membrane by a glyceride-cysteine bond [3]. To investigate the possibility of LevT being a membrane-anchored lipoprotein, the Cys12 residue of LevT was replaced by alanine, and the resultant mutant LevTC12A was assayed for the expression of the P*levD-cat* fusion in various sugars. The results indicated that little change in the expression of the *levD* promoter was caused by the C12A replacement in the LevT protein (Table S3) except when fructose was used to culture the bacteria, which resulted in a nearly 2-fold increase in CAT activity. Notably, none of the LevT/RbsB homologues that are encoded as part of the conserved *levQRST* operon structures found in the six other bacteria that harbor apparent *levQRST* operons possesses a conserved cysteine residue in the N-terminus. When the Cys149 residue in LevT was replaced by alanine, the resultant mutant yielded higher P*levD-cat* activities than the wild-type strain when cells were cultured in glucose or galactose, but little difference was seen in fructose-grown cultures (Table S3). Interestingly, Cys149 of LevT is conserved in the LevT proteins encoded in the *levTSRQ*-like operons of *Lactobacillus johnsonii* NCC533, *Dorea longicatena* DSM 13814 and *Clostridium acetobutylicum* ATCC 824.

**TABLE S3.**  Expression of *levD* promoter:*cat* fusion.

| **Strain** | | **CAT specific activity ± SD on various growth carbohydrates** | | | |
| --- | --- | --- | --- | --- | --- |
|  |  | Glucose | Fructose | Mannose | Galactose |
| ***levQ^+^levT^+^*** | | 2.8 ± 0.4 | 474.0 ± 44.7 | 870.4 ± 85.2 | 9.6 ± 0.6 |
| ***levQ*C161A** | | 114.9 ± 4.3 | 813.9 ± 33.4 | 384.9 ± 28.4 | 191.6 ± 10.8 |
| ***levQ*C188A** | | 8.7 ± 3.4 | 632.8 ± 24.0 | 959.5 ± 130.8 | 29.8 ± 8.7 |
| ***levQ*C296A** | | 8.0 ± 1.0 | 463.1 ± 17.5 | 734.6 ± 33.7 | 71.2 ± 5.9 |
| ***levQ*C336A** | | 3.5 ± 0.9 | 484.0 ± 12.8 | 587.5 ± 54.6 | 15.8 ± 3.0 |
| ***levT*C12A** | 3.1 ± 1.5 | | 840.2 ± 79.2 | 977.0 ± 71.8 | 12.4 ± 1.0 |
| ***levT*C149A** | 8.7 ± 1.2 | | 666.8 ± 17.2 | 1,131.0 ± 53.4 | 44.5 ± 8.6 |

**References**

1. Woodson K, Devine KM (1994) Analysis of a ribose transport operon from *Bacillus subtilis*. Microbiology 140: 1829-1838.

2. Russell RR, Aduse-Opoku J, Sutcliffe IC, Tao L, Ferretti JJ (1992) A binding protein-dependent transport system in *Streptococcus mutans* responsible for multiple sugar metabolism. J Biol Chem 267: 4631-4637.

3. Sutcliffe IC, Tao L, Ferretti JJ, Russell RR (1993) MsmE, a lipoprotein involved in sugar transport in *Streptococcus mutans*. J Bacteriol 175: 1853-1855.
